# Book Reviews

**Published:** 1826-10

**Authors:** 


### CRITICAL ANALYSES.

Quæ laudanda forent, et quæ culpanda, vicissim

Illa, prius, cretâ; mox hæc, carbone, *notamus*.—Persius.

*Transactions of the Medico-Chirurgical Society of Edinburgh.* Vol. II. With Plates.—8vo. pp. 411. Black, Edinburgh; Longman and Co. London. 1826.

Of twenty-seven articles contained in this volume, thirteen only come under the denomination of "papers," the rest being made up of detached cases. Eight of the communications embracing general observations, are by practitioners not resident in Edinburgh; so that the contributions from the practitioners of the northern metropolis amount only to five papers containing general remarks, and nine cases. The cases we shall pass by, throwing such of them as are interesting into the department of our Collectanea; the papers we shall analyse.

*Account of the Exanthematous Ophthalmia, with Observations on its Treatment.* By James Wardrop, Esq. &c.

The object of this paper is to describe a particular form of ophthalmia, which the author asserts has generally been confounded with scrofulous inflammation. As the name implies, this variety of the disease is connected with cutaneous eruptions, some form of which always accompanies or precedes: sometimes it immediately succeeds measles, scarlatina, and other exanthematous diseases; but usually it does not appear till some time after they have subsided.

"The symptoms of the exanthematous ophthalmia are very characteristic; for, besides being connected with eruptions, and confined to young people, the excessive intolerance of light, the enormous secretion of tears, and the relief from forcibly squeezing the eyes, are symptoms quite peculiar. The patient afflicted with this disease can scarcely hold up his head, and, if he is desired to open his eyes, he is affected exactly as if he were looking on a mirror reflecting a bright sunshine, every attempt causing a profuse gush of tears, and being instantly succeeded by a violent and involuntary squeezing of the eyelids, and knitting of the eyebrows. He excludes all light, not only by holding down his head and squeezing the eyelids together, but by pressing a handkerchief firmly on them, or by resting his face against a chair in some dark corner of the room. When in bed, he lies with his face buried in the pillow; a circumstance which, of itself, points out the peculiarity of this inflammation, and distinguishes it from all others.

"The intolerance of light is always most severe in the morning; but in the afternoon it sometimes remits so much as to allow the patient to open his eyes, and see to a very considerable degree, for some hours. The tears, besides being of an extraordinary quantity, are of an acrid, irritating quality, producing violent paroxysms of sneezing, scalding the cheeks, the alæ of the nose, and the lips; so that these become inflamed and swelled, and sometimes covered with pustules and cutaneous ulcerations. The eyelids are also swelled, and have turgid veins on their surface. On trying to force them open, a torrent of tears gushes out; and it is not without occasioning great pain that a small portion of the globe can be exposed. An attempt to get a view of the cornea gives great pain, and it is almost impossible to succeed. The palpebræ, as well as the sclerotic conjunctiva, are but slightly reddened; the vessels appearing as a few distinct trunks, instead of the diffused redness observed in many other inflammations. In general, both eyes are attacked with this disease, though one more violently than the other." (P. 3.)

Along with the local symptoms, there is more or less constitutional disturbance, and the disease remains for many days or weeks; and Mr. Wardrop has known it continue for months, "and even years."

The treatment consists in general rather than in local remedies: indeed, cleansing the eyes with warm water is the only application which ought to be had recourse to at the beginning.

"The general treatment which is commonly necessary for the cure of the exanthematous ophthalmia, consists of first completely evacuating the bowels, and afterwards regulating them; of giving alterative and tonic remedies; and of producing an artificial discharge. Even when this ophthalmia appears in a feeble and emaciated child, it will usually be found that, by the exhibition of purgatives, feculent matter, both unnatural in quantity and of a bad quality, will be evacuated; and, until its evacuation has been effected, other remedies avail little. One grain of calomel with three of rhubarb, given at bed-time, and repeated every other night, four or five times, whilst jalap or senna is taken the alternate mornings, will generally answer the purpose of bringing away the feculent contents of the primæ viæ. But, whenever the quality of the evacuations improves, these medicines must be given with caution; and one dose of the rhubarb with calomel, given only once in six or eight days, and the senna or jalap occasionally, will be sufficient. For, though the greatest benefit will be obtained by evacuating the bowels, violent purging will be found equally prejudicial. When the treatment has been so far advanced, that only one dose of calomel appears necessary in six or eight days, then at this time tonic and stomachic medicines may be advantageously administered. Of these I have found none so generally useful as the carbonates of soda or potass, either given singly, or combined with rhubarb and the bitter infusions. In some instances the mineral acids have been very useful, and also the preparations of iron. Whilst using either of these remedies, much attention is also due to food and habits of life. All wines and malt liquors are particularly hurtful; and the patient should live chiefly on farinaceous vegetables, with but a very small proportion of animal food. The body should not be loaded with clothes, and the head particularly should be slightly covered; protecting the eyes with only a single and narrow fold of black silk, hanging loosely over them, and not wearing a large bonnet. The hair ought to be cut very short; and the greatest advantage will be found from sponging the head and neck with water every morning,—using it at first of an agreeable temperature, and making it colder by degrees; particular care being taken to dry the head well afterwards." (P. 6.)

When the disease has been severe and long continued, it often happens that the patient's general health gradually declines; and, when this takes place, the ophthalmia is apt to return. The seton or pea-issue are said to be very beneficial in preventing a relapse; and accordingly we are advised to employ one or the other, with long-continued attention to the bowels, and the other means usually adopted to invigorate the system.

*Observations on the Nature, Causes, and Treatment of Beriberi.* By WM. HAMILTON, Esq. Surgeon, H.E.I.C.S.

The disease which forms the subject of this paper has been described by various writers, and the author before us adds little to what has already been stated regarding it. It is almost exclusively confined to Ceylon, the Malabar coast, and that part of the country extending from Madras to Ganjam. The symptoms are thus detailed:

" Great debility, with difficulty of respiration, a sense of weight and oppression at the lower end of the sternum, and an almost paralytic state of the thighs and legs, which, soon after the commencement of the attack, became œdematous; as did also the face, and indeed the greater part of the body, with a general sense of coldness over the surface; pulse 120, small, feeble, and intermitting. All these symptoms went on increasing until the death of the patient, which took place within forty-eight hours from the time that I first visited him. A short time previous to his death, he was seized with a violent fit of vomiting, spasms of the abdominal muscles, and increased dyspnœa, which carried him off." (P. 17.)

As in almost all other diseases of tropical climates, bleeding, calomel, and opium, are recommended. We are prevented, however, from entering more fully into particulars at present, because we have received an original Paper on the subject, which we purpose laying before our readers on a future occasion.

*Observations on Chronic Inflammation of the Iris.* By ALEXANDER WATSON, Esq.

This is a very short paper: it refers only to the appearances presented by the eye, no mention being made of the treatment. The phenomena are thus detailed:

"In this disease, the first change observed in the eye is a partial irregularity of the pupillar margin of the iris, at one or more points. This irregularity of the margin of the iris alters, of course, the form as well as the size of the pupil. In some cases it is more dilated, and in others more contracted, than natural. The iris loses its proper colour; and its pupillar margin becomes partially or wholly drawn backwards, in consequence of its partial or complete adhesion to the capsule of the crystalline lens. The motions of the pupil at the same time become impeded, in proportion to the number and extent of the adhesions. In some cases, the adhesion of the iris takes place to a considerable extent, at one point: in other cases, the adhesion takes place, to a smaller extent, at several points, which, in the progress of the affection, by extending, become one continued adhesion, involving the greater part or the whole of the pupil. That part of the iris between the adhesion and the ciliary margin assumes a convex form, by projecting to a greater degree, at this part, towards the cornea, than it does in its healthy state. By this projection, the size of the anterior chamber of the aqueous humour is diminished, and that of the posterior chamber is proportionably increased.

"Where the iris adheres to the capsule of the lens, an effusion of lymph may, in general, be observed, forming the connecting medium. The capsule of the lens generally becomes opaque; and frequently small portions of lymph, and sometimes of pigment, from the posterior surface of the iris, can be seen upon this capsule. In some cases a deposition of lymph takes place upon the inner surface of the cornea, occasioning a dimness and opacity of this part. Vision gradually becomes impaired as the disease advances, till it is quite destroyed. And this last symptom (impaired vision) is commonly the only one by which the patient is conscious that mischief is going on in the eye.

"The progress of chronic inflammation of the iris is remarkably slow and insidious, having been reported, in several cases, to have continued for many years gradually destroying the sight; and that, too, notwithstanding the employment of various remedies to arrest its progress. In some cases it has appeared to take place after an attack of acute inflammation of the eye, and in others after rheumatic complaints." (P. 43.)

The disease above described is regarded as exactly resembling chronic inflammation of the serous membranes in the cavities of the chest and abdomen; and this analogy is supposed by the author to afford a strong proof that the cavities of the aqueous humour are likewise lined by a serous membrane. The appearances of the eye are illustrated by an engraving.

*An Account of the Yellow Fever, as it appeared in the Queen's Regiment, in Barbadoes, in* 1816 *and* 1817. By A. J. RALPH, M.D. Assistant Surgeon to the Regiment.

We have been inundated with accounts of the yellow fever during the last few years, and, as the subject is one of comparatively little interest to the British practitioner, we shall take leave to pass over this paper; by which, however, we by no means wish to insinuate that the account before us is not quite as good as most others. The author is a non-contagionist.

*Observations on the prevailing Opinions respecting Respiration and Animal Heat; with Experiments.* By C. J. B. WILLIAMS, M.D.

In this country, the most prevalent theory on the subject of animal heat, is that which was founded on the discoveries of BLACK, PRIESTLEY, and LAVOISIER, and which was afterwards modified in consequence of the experiments of Mr. ELLIS. According to this doctrine, venous blood contains a quantity of superfluous carbon, which it gathers in the course of the general circulation. To remove this carbon is the office of the lungs, and the evolution is supposed to be effected by a process of secretion, the carbon uniting with the oxygen of the inhaled air, so as to constitute the carbonic acid expired. The other theory originated with LA GRANGE, and according to it the blood, in its progress through the lungs, has oxygen substituted for carbonic acid; the arterial being reconverted into venous blood by the combination of its oxygen with carbon, in the course of the circulation. Now, the object of the first part of the paper is to examine the pretensions of these theories, and show the grounds on which they are respectively founded;—the question is thus argued:

"The chief peculiarity in Mr. Ellis's theory is, that it considers arterialisation as the result of a process of secretion. Now, in several points of view, this doctrine appears objectionable, as being opposed by known facts in chemistry and physiology. In the first place, it is assumed that the pulmonary vessels have the power of isolating and secreting an *elementary* substance, simple carbon, from the principles composing the blood; although such an opinion is at variance with the laws of secretion, as far as they are known. It may be remarked further, that the secretory power, whatever it be, may be influenced by certain affections of the nervous system, as the experiments of modern physiologists sufficiently indicate; and, were the change of the blood in the lungs the result of a secretion, it might be expected that it would be influenced by similar laws. But an appeal to the same experimentalists will prove that the case is otherwise, and that the process of arterialisation is scarcely, certainly not correspondently, impaired by injuries of the nervous system.

"In the next place, if we suppose carbon to be secreted from the exhalants in the lungs, I ask, does any analogy justify the opinion that this carbon can unite with oxygen at the temperature, and in the circumstances, in which they are here supposed to exist? I rather think, that whoever attentively examines those natural processes, in which carbonic acid is formed, will concur with me, if I reply in the negative. It is true that, in some putrefactive processes, carbonic acid may result from the union of the carbon of the putrifying matter with the oxygen of the air at a low temperature; but let it be remembered that the combination is, in such instances, effected by the co-operation of other affinities, and, consequently, that the cases are not parallel. The case under our notice is that of simple, isolated, and consequently *solid carbon,* in its habitudes with atmospheric air. Nor can any aid be derived from the agency of a vital principle, (that omnipotent power, to whose operation the most miraculous effects have been ascribed;) for, according to this theory, the union can only take place after the carbon has been excreted, and consequently removed beyond the sphere of such influence. This is, perhaps, the principal objection against the theory. But this is not all.

That the conversion of venous blood to the arterial state can take place out of the body, by subjecting it to the action of oxygen, is a fact too well established to admit of dispute; yet neither organic structure nor living action can operate here; and the advocates of this theory must therefore resort to some other mode to account for the supposed separation of carbon. This difficulty Mr. Ellis has attempted to remove, by attributing the change to the escape of carbon by *evaporation;* but such an assumption, as it attributes to carbon properties which it has *not,* can hardly be admitted.

"The other theory which I have noticed, is not, at least, liable to these objections. The absorption of oxygen by animal fluids, is by no means singular. Saliva and mucus have a faculty of absorbing oxygen, and again yielding it to other substances. Fourcroy has ascribed a similar property to albumen, which bears closely on our present subject. The simultaneous evolution of carbonic acid gas is rendered probable by the experiments of Macbride, Vogel, Sir E. Home, and Dr. Scudamore, which, although liable to some fallacies, seem sufficient to show its existence in the blood, and the facility with which it may be extricated. I consider this evolution as the result of a chemical displacement, caused by the superior affinity of oxygen for the blood." (P. 93.)

Having endeavoured to show that the change effected on the blood in the lungs consists in the acquisition of oxygen, and the loss of carbonic acid, Dr. Williams next inquires by what power such change is produced? He inclines to the opinion that it arises from a chemical affinity between oxygen gas and the blood; the oxygen, in the process of arterialisation, displacing the carbonic acid from venous blood. He prefers this explanation to that of the modern French physiologists, who ascribe the change to a vital exhalation and absorption: first, because the same change is produced out of the body; secondly, because this change does not cease in animals when most of the vital functions are interrupted; thirdly, because, in the experiments of Le Gallois, when the heart's action had ceased, and there could have been no active exhalation or absorption by the blood-vessels, yet, by inflating the lungs, the arterial hue was communicated to the blood, as far as the carotids; and fourthly, because many animal fluids possess the property of absorbing oxygen, while carbonic acid appears to exist in the blood in a very loose state.

The second part of the paper relates to the theory of animal heat, and, on this subject, our author is convinced that the union of carbon and oxygen, to the extent occurring in the body, is insufficient in itself to account for the constant and uniform temperature of so large a mass of matter, exposed to so many sources of cooling. At the same time, however, he is of opinion that the chemical union of these elements is a principal source of animal heat; but that, as it is only one of many changes "by which animal matters are resolved into simpler principles," so he conjectures that it forms a part only of the means by which the temperature of animals is supported. For the experimental details, we must refer to the volume itself.

*Cases illustrating the Contagious Nature of Erysipelas, and its Connexion with severe Affection of the Throat.* By John Stevenson, M.D.

This paper is in the form of a letter to Dr. Thomson. The most interesting part of it relates to the connexion of erysipelas with a particular form of cynanche. We remember to have heard this pathological fact mentioned by Dr. Chambers, in his Lectures, and have ourselves recently met with some examples of it. We subjoin Dr. Stevenson's account:

"I enclose a short abstract of some of the cases which, in our late conversation, I mentioned to have occurred in my practice here, tending to prove the contagious nature of erysipelas, and to show its connexion with a peculiar and severe affection of the throat, of which I do not recollect to have met with any description. This affection of the throat occurred so frequently in persons who had been much with erysipelatous patients, that I could not doubt their identity; and I came finally to the conclusion, that it was in reality erysipelas of the fauces, spreading occasionally to the adjacent parts in different directions. The febrile symptoms by which it was ushered in were generally severe, even in the milder cases; very full and frequent pulse; severe pain of head and back; restlessness, and great heat of the surface. The period at which the affection of the throat came on, after the accession of the fever, varied from the second to the sixth day. It commonly began with a red or purplish blush, more or less extensive, over the velum pendulum and uvula, accompanied with very little tumefaction, but with considerable pain in swallowing: often, after a few days, excoriation of the inflamed surface followed, with superficial ulceration, which at times soon healed, but at other times spread, and discharged a good deal of purulent matter. In many cases the disease terminated without extending farther than the parts mentioned, but in a few it spread to the larynx, producing a state of respiration very like that of idiopathic croup: in others, it extended to the pharynx and œsophagus. When the last became affected, fluids, and even solids, could be partially swallowed without much apparent difficulty; but, after a few seconds, pain was felt in the course of the gullet, an inverted action began, and they were wholly or partially returned to the mouth. In some protracted cases, glandular swellings appeared in the neck, which suppurated externally.

" This disease was readily distinguishable from cynanche tonsillaris, by the want of swelling, by the redness being more diffused, and by the pyrexia being generally greater than could have been expected from the degree of local affection. From croup, it was distinguishable by the larynx being affected in a small proportion only of the cases, by the inflammation not commencing there, (at least, in any case which came under my observation,) and by the age of the patients. From scarlatina, it was distinguishable by the absence of cutaneous eruption, and by its attacking persons who had already had that disease.

" Copious and repeated bleeding, with brisk purgatives, and, in every case where the throat was severely affected, the application of a large number of leeches to the neck, appeared to me to be the mode of treatment which was most successful. All the cases that came under my management, both of the common erysipelas and of the affection of the throat, were treated in this manner. I have stated the event of all those detailed, and it affords a fair specimen of the general results of the cases which came under my care." (P. 128.)

(To be continued.)

*The Anatomy of the Fœtal Brain; with a comparative Exposition of its Structure in Animals.* By Frédéric Tiedeman, Professor in the University of Heidelberg, Member of the Academy of Sciences of Munich and Berlin, &c. &c. *Translated from the French of* A. J. L. Jourdan, by William Bennett, m.d. *To which are added, some late Observations on the Influence of the Sanguineous System over the Developement of the Nervous System in general.* Illustrated by fourteen Engravings.—8vo. pp. 324. Carfrae and Son, Edinburgh; Longman and Co. London. 1826.

The anatomy of the brain, in all animals, and under almost every conceivable circumstance of health and disease, has occupied the attention of many of our most distinguished brethren, both at home and abroad, during the last twenty years; and there is reason to suspect that the merely mechanical part scarcely admits of improvement: the description may vary, but that which is described remains the same. The very distinguished author of the present work appears to have struck out for himself a line of investigation, in which the ground has been less trodden—the peculiarities of the Fœtal Brain. There is every appearance of candour and good faith in the manner in which the details are given, and we nothing doubt their accuracy: at the same time, the nature of the investigation places great difficulties in the way of verifying his observations, and thus unavoidably detracts from the general interest of the subject.

Every source whence light may be admitted upon the obscurity of the nervous system, ought doubtless to be appreciated, and our attention has been too exclusively confined to the brain in its adult and perfect state. To what extent an acquaintance with the manner in which nature builds up the cerebral fabric may elucidate its operations, would not be easy to determine; but, as physiology must always take anatomy for its basis, no one can deny the possibility of some assistance being gained. Perhaps, the first step towards erecting a more permanent superstructure, is the removal of that which is flimsy and unsubstantial: thus far the labours of Tiedeman have already been negatively available, as they have shown that many opinions, which have heretofore passed current with regard to the structure of the brain, are the offspring of imagination. A curious and interesting fact, of a positive character, which he has proved by comparative anatomy, is that the brain of the human fœtus, in its progressive development, passes through all the principal degrees of organisation, at one or other of which it is permanently arrested in the vertebral animals; thus showing that nature follows a regular plan in the evolution of the brain, stopping short at a certain point, or contributing to the further perfection of the organ, according to the rank which the animal is destined to hold in the scale of existence.

The idea of GALL, that the medullary matter is the product of the grey, is entirely disproved by the result of these investigations; and some further interesting facts relative to the production of the different portions of the nervous system, are given in the Addenda placed at the end of the work, containing the views of M. SERRES. According to this physiologist, the spinal marrow is produced by the intercostal arteries, the cerebellum by the vertebral, and the brain by the carotids; so that the question of the pre-existence of the different parts is contained in that of their arteries: such order being, first, the spinal cord; secondly, the crura cerebri and tubercula quadrigemina; and, lastly, the cerebellum.

But it is in vain to enter upon any analysis of such a work: to the general reader it is not of sufficient interest; while the anatomist would not, or ought not, to be contented with any review, however extended.

The translation is given in good language, and the plates are distinct and well executed. We must remark, however, of Fig. 7, Plate 1, that it has a ludicrous resemblance to a human head; the chin, mouth, nose, and right eye, are quite distinct: this ought to be avoided, as it gives the idea of the parts being caricatured.

The following quotation will show the general views of the author, and the manner in which his meaning has been rendered by his translator.

"In my method of viewing the subject, there are but two paths, hitherto little frequented, which can lead to the knowledge of the structure of the brain: these are comparative anatomy, and the anatomy of the fœtus.

"Comparative anatomy unveils to us the origin and successive development of the brain and nervous system, from the most simple animals, up to man, the most complicated. There is no set of organs, in the formation of which we find so perfect a gradation from the simple to the compound, as in the cerebral and nervous system: in fact, this system is established on an uniform plan in the whole animal scale. So, in studying the gradual complication of the structure of the brain in animals, can we have a clear idea of the complex organisation of this viscus in man, and at length succeed in comprehending its assemblage and relations.

"Though the moderns have well estimated the utility afforded by comparative anatomy in this subject, they have, however, profited little from the advantages placed before them. If we take a rapid view of the great work of Gall, we find one idea reigning through the whole,—that it is necessary to study the structure of the brain and nervous system, gradually ascending from the more simple animals, up to man. But what has he done? He has merely described and represented, respecting the nervous system of animals, the nerves of the caterpillar, the brain and spinal marrow of the chick, and of some of the mammiferous animals; yet even his work on this subject is not exempt from errors. To set out from so small a number of data, in order to arrive at general conclusions respecting the structure of the brain and nervous system, would indeed render the question still more complicated than it really is, in place of throwing one salutary ray of light upon it. We should consider these partial works but as materials of a grand edifice; but while we employ them as elementary principles for general propositions, we cannot fail to be led into new errors. No axiom relative to any point of anatomy or physiology can be established, unless skilfully deduced from all the facts and observations on the object in question.

"As, by the study of the brain and nervous system of animals, we can alone arrive at the knowledge of the gradation which the former undergoes in its formation and progressive complication, so also shall we have need of a comparative psychology, to conceive the uses and manner of action of each portion which composes this mass. We must observe attentively the phenomena of cerebral action, from animals the lowest in the scale up to man, and then compare them with the structure of the organ itself. This comparative study of the actions and organisation of the brain in the different animals, will dispel the cloud from o'er the functions devolving on its separate parts; a knowledge to be derived from no other means than those already mentioned. It is a general truth, recognised at present, that the cerebral functions of animals become more numerous and diversified, according as their brain and nervous system possess a more complicated structure; and we also know that the nerves of sense, and their roots in the brain, are more voluminous, according as the organs of sense are better developed. We cannot, then, doubt the existence of a perfect relation, an intimate connexion, between the acts of intelligence in animals, and the structure of their brain.

"In following this method, we may arrive at the knowledge of the function of each part of the encephalic mass; but the intimate essence, the proximate cause of all these phenomena, will still remain shadowed from our view by the thick veil of darkness which covers them. Is the mind similar to the matter of the brain? or, rather, are they different things; and is then the brain in some degree the organ, the material instrument of the mind? Such problems, philosophers and physiologists have not yet been able to resolve, nor never will. I repeat it, anatomy, physiology, and psychology, may teach us the structure of the brain, and the action or function of its different parts; but they will never unveil to us the essence of this action:—Such is my opinion." (P. 2.)

---

*The Anatomy of the Brain, with a general View of the Nervous System.* By G. SPURZHEIM, M.D. of the Universities of Vienna and Paris; Licentiate of the Royal College of Physicians in London. *Translated from the unpublished French MS.* by R. WILLIS, Member of the Royal College of Surgeons in London. With eleven Plates.—8vo. pp. 234. London: S. Highley, 1826.

WE have been in the habit, in this country at least, of speaking of GALL and SPURZHEIM conjointly, but, from the tenor of the present work, it is pretty clear that the latter would be better pleased to have the name of his colleague dropt. It appears that he attended Dr. Gall's lectures in 1800, and studied under him till 1804, when he became associated with his master, and soon after began his travels; since which time he seems to have led rather a wandering life, sometimes in Germany,—sometimes in France,—sometimes in England,—and at all times seeking for proselytes. In the first instance the works of the two anatomists appeared as the results of their common labours; but now we are told, that "history will assign to each his share in the works that have issued under their joint names:" and again, "the works which Dr. Gall has published in his own name, fix the extent of his phrenological knowledge." Now, we suspect that history, if it meddle with the matter at all, will be somewhat puzzled between the rival Doctors: for, as Dr. Gall taught Dr. Spurzheim, and as certain volumes were published in which his name stands first, we would presume that he had some share in the discoveries which they contain. But, on the other hand, it would seem to be implied in the sentence above quoted, that Dr. Gall had nothing to do with the works published jointly, as those given to the world in his own name *fix the extent of his phrenological knowledge.* What share these gentlemen have had in the discoveries to which they respectively lay claim, is best known to themselves, and will probably remain so, unless "history" be very much at a loss for something to do.

Their productions may be viewed in two lights—as anatomical, and as physiological. With regard to the former, we believe there is little to be found in their writings that had not been previously described by VIEUSSENS, MONRO, VICQ D'AZYR, and REIL: nay, even with regard to the manner of dissecting the brain, the first of these employed the method of scraping, and the last that of hardening the parts in spirits, so as to admit of their being more minutely unravelled. Many of the descriptions claimed by Gall and Spurzheim may be found in papers by Reil, published in GREN's Journal so early as 1795, being anterior to the earliest period at which Gall lectured. With respect to their physiology, setting aside the idea of a general connexion between the intellectual functions and the development of the brain, both in man and the lower animals, which is not peculiar to them; and referring to that which is especially their own,—viz. the fixing of the precise localities of their five-and-thirty "organs," we regard it as one of the bubbles of the day, fit only for grown-up children and would-be philosophers.

The work is divided into nine sections: the first contains general reflections on the Nervous System; the second, the division of the Nervous "Apparatuses;" the third relates to the Nerves of Voluntary Motion, and of the External Senses; and the fourth to the best manner of examining the Structure of the Brain. Dr. Spurzheim's method is unquestionably preferable to that more usually adopted: we subjoin the most important part of the description.

"Our physiological views do not, it must be evident, allow us to go on cutting the brain into slices: this procedure, indeed, ought rather to be entitled a destruction, than an anatomical demonstration of the cerebral structure: it is precisely as though one should pretend to dissect a leg or an arm, by slicing down these members transversely; or to show the structure of the thoracic and abdominal viscera, by treating the trunk in a similar manner, and giving names to the appearances exposed after each successive slice. We commence our dissection at the place where the proper cerebral masses are added to the nervous parts already described; we trace them in their continuations, and in their connexions mutually, and with the nerves of the five senses and of voluntary motion: in short, we proceed in the dissection of the brain in a manner precisely analogous to that which is followed in the anatomical demonstration of the other parts of the body.

"Besides the above general anatomical principle as regards procedure, it is important to know that, on account of their extremely delicate organisation, the structure of several cerebral parts may be more easily and clearly exposed by means of scraping, than by cutting. This is the reason why I frequently prefer the handle to the blade of the scalpel, for removing parts that cover those whose course I would show: for instance, the passage of the pyramidal bodies across the annular protuberance; the continuation of the anterior commissure through the striated bodies into the middle lobes of the brain, and of the anterior pillars of the fornix, onwards to the mammillary bodies and interior of the thalami.

"The brain should be removed from the cranium, care being taken not to tear the crura at the superior edge of the annular protuberance, (an accident that is very apt to occur,) nor to injure the medulla oblongata at the lower edge of the same part; and to cut the spinal mass so low down as to obtain, besides the entire medulla oblongata, the upper part of the true spinal cord. The brain, thus freed from the skull, is then to be put into a plate, with the basis uppermost. The cerebellum and medulla oblongata, having lost the support of the bone, now fall backwards. In this position, all the appearances presented by the base of the brain are visible. Having considered the cranial nerves in the manner described in the preceding section, the structure of the true cerebral masses is to be examined, commencing with that of the cerebellum." (P. 103.)

The fifth and sixth sections are devoted to the Cerebellum and Brain; in the seventh, the Commissures are explained; and in the eighth, the communication of the nervous parts with each other; while the ninth and last treats of the connexion between certain points in anatomy and physiology.

The whole presents a compendious view of the opinions of Gall and Spurzheim, in a more accessible form than their large works; and it is but justice to add, that the engravings (eleven in number) are very beautifully executed.

---

*Illustrations of Phrenology, in connexion with the Study of Physiognomy.* By G. Spurzheim, m.d. &c. *Part I. Characters.* With thirty-four Plates.—London, 1826.

This is a very *pretty* book, exceedingly well fitted for the drawing-room, where it may be useful in promoting conversation when it begins to flag. Young ladies, of phrenological propensities, may have an opportunity of examining the "organs" of the most distinguished personages of history, "from Macedonia's madman to the Swede," whose high and singular forehead sufficiently evinces him to have been a "striking" character. We notice the work merely as being by the same author, and in some degree connected with the preceding.

---

*An Exposition of the State of the Medical Profession in the British Dominions; and of the Injurious Effects of the Monopoly, by Usurpation, of the Royal College of Physicians in London.*—8vo. pp. 373. London: Longman and Co. 1826.

The object of this work is to prove that all the abuses and evils in the present state of medical practice, are solely to be attributed to the Royal College of Physicians in London.

Reform, indeed, is the order of the day: physicians, surgeons, and apothecaries, are severally protesting against the monopolies of their respective corporations. Into these disputes we have not entered, and mean not to enter; firmly persuaded that, in the present state of excitement, public feeling requires no additional stimulus, and that these, like other *fermentations,* will end in effecting purification to a greater or less extent; while the feculencies which caused the turmoil, will sink to their natural level, as soon as the process is completed.

The volume before us contains references to every point of interest, and to many things of no interest whatever, connected with the history of the College; and it would appear, from the evidence it affords, that jealousies and discontents, similar to those which have brought the present "Exposition" to light, have very frequently given occasion to similar productions, without any beneficial results; and we are inclined to think the similarity will hold good in this respect also. The documents alluded to are very numerous, and to the general reader totally destitute of interest, consisting principally of cases, decisions, and questions which have been at issue between the licentiates, surgeons, and apothecaries, on the one hand, and the fellows on the other.

According to our author, the sole cause of all the evils in medicine is the existence of the College of Physicians,—the *unicum remedium,* its abolition. Such an event is a mere chimera. Even the members of the College of Surgeons begin to perceive that they cannot abrogate a royal charter by making speeches, and content themselves with the more rational attempt to enforce the privileges granted them by the very deed they would have annulled.

We repeat, however, that it is not our intention to enter upon these subjects; and the only part of this volume which we think of sufficient interest for quotation, relates to the relative proportion of the members of the different branches of the profession in London, as compared to Paris.

"According to printed official lists, there are this year (1825,) forty-five fellows, two candidates, three inceptor candidates, and 124 licentiates, who practise in London and seven miles round: in all, 174 physicians, to supply 1,200,000 inhabitants* with medical assistance, or one to every 7000!

"Upon similar authority, it appears that, of about 5,650 members of the College of Surgeons in London, in 1825, upwards of 800, or one in seven, practise in the metropolis. To this number, for the reasons which shall be assigned in the next paragraph, 200

* "The real number is computed to be nearer a million and a half; but we wish to under-rate rather than over-rate."

may be added; being in all 1000, or at the rate of one surgeon to every 1,200 inhabitants.*

"The printed list of the Society of Apothecaries in London, in 1825, contains about 475 members, of whom not quite 300 reside in the metropolis and seven miles round. But, for the following reasons, this must be considered but as a very small proportion of all the apothecaries exercising their profession in the metropolis:—1. Upwards of a century ago, their number exceeded a thousand. 2. By the terms of the Acts of Parliament which have recently been passed respecting apothecaries, all those who were exercising the profession in London, previous to 1815, without having been incorporated, or undergone examination, were allowed to continue their functions. This description of persons is very numerous, and their names are not inserted in the list of the Society. 3. All the medical officers of his Majesty's army and navy, and of the East-India Company's service, are entitled to practise as surgeons and apothecaries in any part of the British dominions. Many of them avail themselves of this privilege by settling in the metropolis, and their names are not to be found in any list. We are then, probably, much within the mark, when we estimate the whole number of apothecaries in London, derived from all of these sources, at 2000; being at the rate of one apothecary to every 600 inhabitants.

"Thus, in London, the physicians are to the surgeons as one to six; to the apothecaries, exclusive of the chemists and druggists, as one to twelve; to both united, as one to eighteen!

"The number of chemists and druggists in the metropolis, we estimate at about 300.

"In Paris there were, in 1822, 600 physicians; being, on a population of 800,000, in the proportion of one physician to 1,333 inhabitants, or five times greater than in London.

"The same year, there were in Paris 128 surgeons; being one to 6,250 inhabitants, or four-fifths less than in London.

"The number of apothecaries in Paris, in 1822, was 181; being one to 4000 inhabitants, or five-sixths less than in London.

"In Paris, then, the physicians are nearly five times as numerous as the surgeons, more than three times as numerous as the apothecaries, and twice as numerous as both.

"Thus, the ratio which the physicians of Paris bear to its surgeons and apothecaries united, is to the ratio which the physicians of London bear to its surgeons and apothecaries united, as *thirty-six to one!*

"Consequently, if in Paris the three branches of the profession bear a due proportion to the demands of the inhabitants for medical, chirurgical, and pharmaceutical aid, and if the relative

* "We are assured that the surgeons in London amount to 1,500; but, not having the immediate means of an accurate estimate, we choose to adhere to the lesser number, which will sufficiently justify our conclusions."

demands of the inhabitants of both capitals for the aid of these several branches be similar, the actual excess of surgeons and apothecaries over physicians in London is as thirty-six to one. This is, in fact, the case; and the excess of surgeons and apothecaries beyond the due proportion of these branches has been produced by the necessity of procuring medical aid from other than the ordinary sources, occasioned by the undue limitation of physicians under the College monopoly.

"Taking the three branches of the medical profession in Paris, and supposing the distribution of them in that capital to be the proper standard,—viz. 600 physicians, 128 surgeons, and 181 apothecaries,—they are united about 900, or at the rate of one to every 900 inhabitants; whilst in London, if the computation of 174 physicians, 1000 surgeons, 2000 apothecaries, and 300 chemists and druggists, be correct, the total number is 3,474, or at the rate of one to every 345 inhabitants. In Paris, then, under a due distribution of the three branches, the expense of maintaining each individual engaged in the profession is divided among nine hundred persons, whilst in London it is shared among 345; the actual expense to each inhabitant of the latter being nearly treble the expense to each inhabitant of the former city." (P. 6.)

---

*Principles of Dental Surgery: exhibiting a new Method of Treating the Diseases of the Teeth and Gums, especially calculated to promote their Health and Beauty; accompanied by a general View of the present State of Dental Surgery, with occasional References to the more prevalent Abuses of the Art. In two Parts.* By LEONARD KOECKER, Surgeon-Dentist, Doctor in Medicine and Surgery; Member of the Medical and Linnæan Societies, and of the Academy of Natural Science of Philadelphia, &c. &c.—8vo. pp. 445. London: Thos. and George Underwood. 1826.

To have a work extending to 445 pages on the Diseases of the Teeth, is a sign that there are some professing the art of the dentist who are determined not to be behind their brethren in other departments of surgery. In this country, those who select particular branches are always looked upon with some degree of suspicion; and assuredly there is more quackery among the oculists, aurists, and dentists, (not to mention chiropodists,) than among those who practise medicine or surgery *generally*. Of late years, dentists have sprung up in every corner,—which may account for so many people having bad teeth: at least, we are fully convinced that there is no department of surgery in which injudicious interference so often takes place, or gives rise to more inconvenience and suffering.

The author of the work before us is a German, who practised for many years in Philadelphia; and, in perusing his book, it is but fair to keep in mind that he is a foreigner, not perfectly acquainted with our language or our usages; and we would, moreover, recommend to our dentists to forgive the unceremonious manner in which their pretensions are treated: the rather, as we believe the censures bestowed upon them to be in general deserved. The volume is one, indeed, from which those interested in this branch of the art will derive much information, and shows Mr. Koecker to be a man of good medical education, as well as of considerable research.

We would express a favourable opinion of the work, but by no means without qualification; for there are many points of which we cannot judge, not possessing sufficient practical acquaintance with this department of the healing art. We can perceive, too, that there is occasional repetition, and that there are too many cases minutely detailed. Here and there we meet with passages which excite a smile. For example, the author strongly recommends the operation of extracting teeth, which, however, he at the same time regards as so unpleasant to the *operator*, that, "had Peter the Great himself been a professional dentist, he is strongly inclined to think that his sanguinary passion for the pastime of extracting teeth would have been soon extinct." Whatever the Czar might have done in that capacity, Mr. Koecker has entered into the subject very fully and minutely, treating even "of the moral means subservient to the due performance of the operation of extracting teeth." In this chapter he points out, with good sense, the impropriety of ever having recourse either to force or deception in the cases of children.

In the course of the work the following subjects are discussed:—

1st. Of the natural history of the teeth and their relative parts: that is, of the gradual formation, and various structures and functions, at different periods of life, of the teeth, the gums, the sockets, the periosteum, and jaw-bones, in their healthy state.

2d. Of the different diseases to which the teeth and their relative parts are subjected; their symptoms, together with their remote as well as proximate causes, at the different periods and stages of their formation and structure.

3d. Of the connexions, sympathies, and influences of the teeth, the gums, the sockets, the periosteum, and the maxillary bones, generally and individually, one upon another, in their sound and morbid state; and particularly those of the first set upon the permanent teeth.

4th. Of the influences and effects of those parts in their healthy and diseased states, as well as of those of the whole constitution when in health, or when labouring under any general or local disorder upon the teeth, and the other parts immediately connected with them, at different periods of their formation and structure.

5th. Of the various medical and surgical remedies which the art affords; their judicious exhibition; and the opposite effects produced, as well immediately as permanently, by the application of skilful or improper treatment.

6th. Of the surgical apparatus and mechanical means for the proper application of the above remedies and operations in dental surgery, added to a general scientific knowledge of mechanism, and the various collateral mechanical arts intimately connected with its practice.

---

